# Knockdown of Myostatin Expression by RNAi Enhances Muscle Growth in Transgenic Sheep

**DOI:** 10.1371/journal.pone.0058521

**Published:** 2013-03-20

**Authors:** Shengwei Hu, Wei Ni, Wujiafu Sai, Ha Zi, Jun Qiao, Pengyang Wang, Jinliang Sheng, Chuangfu Chen

**Affiliations:** 1 College of Animal Science and Technology, Shihezi University, Shihezi, China; 2 Key Laboratory of Agrobiotechnology, Shihezi University, Shihezi, China; Johns Hopkins University School of Medicine, United States of America

## Abstract

Myostatin (MSTN) has been shown to be a negative regulator of skeletal muscle development and growth. MSTN dysfunction therefore offers a strategy for promoting animal growth performance in livestock production. In this study, we investigated the possibility of using RNAi-based technology to generate transgenic sheep with a double-muscle phenotype. A shRNA expression cassette targeting sheep MSTN was used to generate stable shRNA-expressing fibroblast clones. Transgenic sheep were further produced by somatic cell nuclear transfer (SCNT) technology. Five lambs developed to term and three live lambs were obtained. Integration of shRNA expression cassette in three live lambs was confirmed by PCR. RNase protection assay showed that the shRNAs targeting MSTN were expressed in muscle tissues of three transgenic sheep. MSTN expression was significantly inhibited in muscle tissues of transgenic sheep when compared with control sheep. Moreover, transgenic sheep showed a tendency to faster increase in body weight than control sheep. Histological analysis showed that myofiber diameter of transgenic sheep M17 were bigger than that of control sheep. Our findings demonstrate a promising approach to promoting muscle growth in livestock production.

## Introduction

Myostatin (MSTN), a member of the transforming growth factor beta (TGF-β) superfamily, functions as a negative regulator of skeletal muscle development and growth. MSTN gene knockout mice have about a doubling of skeletal muscle weights throughout the body as a result of a combination of muscle fiber hyperplasia and hypertrophy [1]. Natural gene mutations of MSTN have also been reported in some cattle breeds [2]–[4], sheep [5], dogs [6] and human [7]. These animals show a double-muscled phenotype of dramatically increased muscle mass, and still viable and fertile [2]–[7]. These findings have suggested that strategies capable of disrupting MSTN function may be applied to enhance animal growth performance.

RNA interference (RNAi) is a process of sequence-specific, post transcriptional gene silencing, which has been used to analyse gene function and develop novel animal models [8]. Several groups, including us, produced transgenic RNAi mice which showed a gene knockdown phenotype that was functionally similar to gene knockout [9], [10]. The ability to generate RNAi transgenics is especially significant for livestock animal for which stem cells have yet to be derived. Recently, transgenic RNAi zebrafish with MSTN knockdown were successfully produced, which resulted in giant- or double-muscle in transgenic zebrafish 11,12. These findings suggest that animal growth performance could be improved by knocking down MSTN using RNAi technology.

In this study, we investigated the possibility of using RNAi technology to generate transgenic sheep with a double-muscle phenotype. Our results showed that shRNA targeting MSTN effectively inhibited endogenous MSTN expression in transgenic sheep. Moreover, transgenic sheep showed a tendency to faster increase in body weight than normal controls. Our study provide a promising approach for the production of transgenic double-muscle animals.

## Materials and Methods

### Ethics Statement

All experiments involving animals were conducted under the protocol (SU-ACUC-08032) approved by the Animal Care and Use Committee of Shihezi University. All sheep involved in this research were raised and breed followed the guideline of Animal Husbandry Department of Xinjiang, P.R.China.

### Plasmid Construction

**Figure 1:**
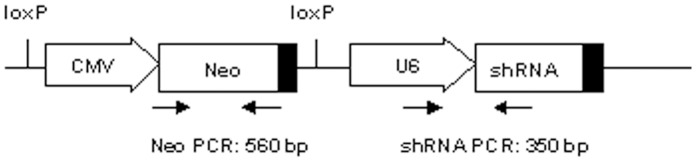
Schematic illustration representing ploxP-shMSTN3 vector used in this study. Loxp: recombination site of Cre recombinase for bacteriophage P1; CMV: CMV promoter; Neo: neomycin gene; U6: polymerase III U6-RNA gene promoter, shRNA: short hairpin RNA. Arrowhead indicated localization of the primers specific for shRNA expression cassette and Neo gene. The size of the PCR amplicons is indicated.

shRNAs targeting sheep MSTN were reported in our previous reports [13]. The shMSTN3 (5′-CAAAGATGCTATAAGACAA-3′) targeted the first exon of sheep MSTN gene. The shMSTN3 expression cassette was amplified using primers U6-F (5′-AGT AGT TGC CAG GAT CAC CGT GC-3′) and U6-R (5′-CCT AAT GAG TG A GCT AAC TCA CA-3′), and then cloned into BamH I and SwaI site of ploxP vector to generate ploxP-shMSTN3 (Figure 1).

### Cell Culture, Transfection and Selection

China Merino sheep fibroblast cells (SF) were isolated and cultured as previously described [14]. 2×10^5^ cells per well were seeded in 12-well plate and cultured in fresh DMEM without antibiotics to achieve 80–90% confluency on the day of transfection. The cells were then transfected with 1.8 µg/well of ploxP-shMSTN3 vectors using Lipofectamine 2000 (Invitrogen) according to the manufacturer’s protocol. After 48 h transfection, cells were split into 100 mm dish at an appropriate dilution for G418 selection (500 µg/ml). Single G418-resistant colonies were obtained after 14 days of selection.

### Construction of Transgenic Sheep by Somatic Nuclear Transfer

Transgenic fibroblast colonies (TF-s2 and TF-s19) were used to construct transgenic sheep. Sheep nuclear transfer (NT) was performed as described reports [15], [16]. Briefly, ovaries were collected from a local abattoir and transported to our laboratory within 4 h after slaughter. Cumulus-oocyte complexes (COCs) were aspirated from 2 to 5 mm follicles with PBS (containing 5% FCS) by using a 5 ml syringe fitted with a 20-gauge needle. The COCs were cultured in maturation medium at 38.5°C in a humidified atmosphere for 22 h. Cumulus cells were removed by exposure to 1 mg/mL hyaluronidase. Oocytes with a first polar body were enucleated manually in the presence of 7.5 µ*g*/ml of cytochalasin B. A single intact donor cell was injected into the perivitelline space and placed adjacent to the recipient cytoplasm. After injection, reconstructed embryos were transferred into an electrical fusion chamber overlaid with Zimmermann’s fusion medium. Cell fusion was induced with two direct current pulses (1.0 kV/cm, 60 µs, 1 s apart). All fused reconstructed embryos were further activated in 5 µM ionomycin for 4 min, followed by exposure to 1.9 mM 6-dimethylaminopurine in synthetic oviduct fluid with amino acids (SOFaa) for 4 h. Following activation, reconstructed embryos were transferred and cultured in SOFaa. 429 embryos at the 2- to 4-cell stages were surgically transferred into 45 synchronized recipient ewes (7–12 embryos per recipient). Pregnancies were monitored by ultrasound scanning using a trans-abdominal linear probe every two weeks until days 90. Normal control sheep were produced by normal sexual reproduction.

### PCR Analysis

Genomic DNA was isolated from ear biopsy of each lamb using TIANamp Genomic DNA kit (Tiangen Biotech, China). Transgene integration was identified by two independent PCR assays. PCR was performed on 20 ng of genomic DNA using specific primers (Figure 1) for Neo (Neo-F: 5′-ATT CGG CTA TGA CTG GGC ACAC-3′; Neo-R: 5′-CCA GAA AAG CGG CCA TTT TCCA-3′) and for shRNA expression cassette (U6-F: 5′-AGT AGT TGC CAG GAT CAC CGT GC-3′; U6-R: 5′-CCT AAT GAG TGA GCT AAC TCACA-3′). PCR reaction consisted of 95°C for 4 min; 30 cycles at 95°C for 35 s, 58°C for 30 s and 72°C for 40 s; an extension at 72°C for 10 min. PCR products were analyzed by gel electrophoresis.

### shRNA Expression Analysis

Biceps brachii muscle tissues were obtained by surgical biopsy from transgenic and control sheep. shRNA expression was identified by RNase protection assay. Small RNAs were isolated from muscle tissues by using the mirVana miRNA isolation Kit (Ambion). The ^32^P-labeled RNA probes (29 nt) were generated by in vitro transcription using the mirVana miRNA Probe Construction Kit (Ambion). A DNA oligonucleotide under T7 promoter control, which was reverse complement with the target RNA, was used for in vitro transcription. Protection assay carried out using the mirVana miRNA Detection Kit (Ambion) according to kit manufacturer's instructions. The protected RNA probe was detected by autoradiography.

### Western Blot Analysis

Protein extracts were taken from transgenic and control biceps brachii muscles, and then were analyzed by western blot analysis as previously described [13]. A primary rabbit anti-MSTN antibody (1∶1000 dilution) (Sigma-Aldrich) and anti-GAPDH (Sigma-Aldrich) were used in the western blotting. Band intensities were estimated by densitometry and corrected by the respective GAPDH band intensities.

### Real-time RT-PCR Analysis

Total RNAs were isolated from biceps brachii muscle tissues using Trizol (Invitrogen) according to the manufacturer’s instructions. The primer sets were used for amplifying MHCII (MCF: 5′-AAC GAT ACC GTG GTT GGG-3′; MCR: 5′-CAG CAC GCC GTT ACA CCT-3′), MyoD (MDF: 5′-GCG GAT GAC TTC TAT GAT GACC-3′; MDR: 5′-GTG CAG CGT TTG AGC GTCT-3′), Myogenin (MF: 5′-AAG CGG AAG TCG GTG TCTG-3′; MR: 5′-ATT GTG GGC ATC TGT AGG GT-3′) and Smad2 (sMF: 5′-GGG ATG GAA GAA GTC AGC-3′; sMR: 5′-ATG GGA CAC CTG AAG ACG-3′). Real-Time PCR (Stratagene MX3000P) was carried out using SYBR Green (TaKaRa Biotech, Dalian) following the manufacturer’s protocol. The PCR thermal cycle reactions consisted of denaturation at 95°C for 4 min followed by 45 cycles at 95°C for 15 s, 60°C for 1 min. Cycle threshold (Ct) values were normalized to GAPDH, and comparative quantification of mRNA was done by the ΔΔCt method.

### Growth Evaluation and Muscle Histological Analysis

Control sheep (n = 3, male) and three transgenic sheep (male) were weighted at 1, 20, 40, 60 and 90 days after birth. The transgenic M17 showed a fastest increase in body weight and was used for further muscle histological analysis. Biceps brachii muscles were isolated by surgical biopsy from transgenic M17 and control sheep (n = 3). The muscle tissues were fixed in formol for 10 h followed by routine paraffin sectioning and Haematoxilin/Eosin staining. Myofiber diameter and number were determined as previously described [17]. Five representative images (one central and four peripheral) were captured from muscle sections, compounding to 0.7 mm^2^. Myofiber diameter and number were measured with Scion Image software at three independent pictures. Fiber size-distribution diagram was generated and showed as percentage of 500 fibers analyzed. Mean diameter of sheep myofibers represented average value of 500 fibers.

### Statistical Analysis

Expression levels of MHCII, MyoD, Myogenin and Smad2 were analysed statistically by one-way ANOVA and Tukey's test. Data were representative of three independent experiments performed in triplicate. Differences were considered significant at P<0.05.

## Results

### Production of Cloned Sheep

The positive cells were selected as donor cells for constructing cloned embryos by SCNT. The cleavage and blastocyst development rates were 74.4% (131/176) and 14.2% (17/120), respectively. In total, 429 embryos at the 2- to 4-cell stages were transferred to the oviducts of 45 recipient sheep (Table 1). Pregnancy rates were 24.4% (11/45) at forty-five days after embryo transfer. Six recipients were spontaneously aborted and five recipients developed to term. Five lambs were named M17, M18, M21, M23 and M24, respectively. M21 and M24 died 1 h and 22 days after birth, respectively. M17, M18 and M23 have survived for more than six months.

**Table 1:** Summary of SCNT results.

No. embryos transferred	No. recipient sheep	Pregnancy rates	Livebornlamb	No. lamb alive over six month	Cloning efficiency (%)
429	45	24.4% (11/45)	5	3	1.2%

### Evaluation of MSTN Knockdown in Transgenic Sheep

**Figure 2:**
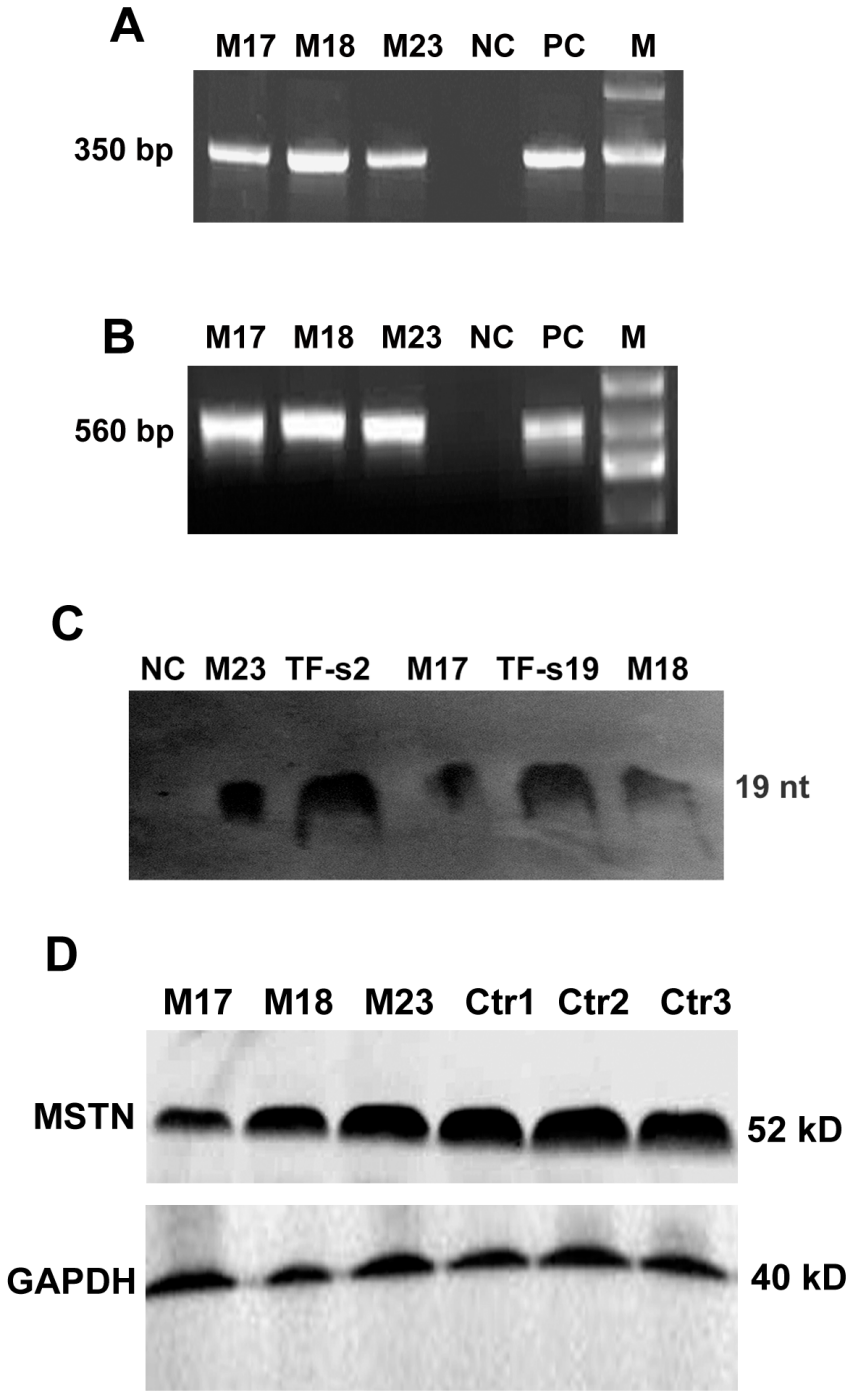
Transgene integration and expression analysis in three cloned lambs. PCR analysis using primers specific for the shRNA expression cassette (A) and Neo (B). (C) Expression of the shRNA targeting MSTN in muscle tissues of transgenic sheep M17, M18 and M23. Negative control (NC): control sheep; positive control (PC): ploxP-shMSTN3 vector. M: Maker; TF-s2: Transgenic cell clone TF-s2; TF-s19: Transgenic cell clone TF-s19. (D) Western blot analysis of MSTN protein expression in muscle tissues of transgenic sheep M17, M18 and M23 and control sheep (Ctr1, Ctr2 and Ctr3).

Genomic DNAs were prepared from the ear tissues of transgenic sheep M17, M18 and M23. PCR was used to detect the presence of the transgene. All the three cloned sheep were positive for the shRNA expression cassette and for Neo gene, respectively (Figure 2A, 2B).

To determine whether shRNAs targeting MSTN were expressed in muscles of transgenic sheep, shRNA expression was analyzed by RNase protection assay. As shown in the Figure 2C, shRNA expression was detected in the muscles of three transgenic sheep, whereas not in the muscles of control sheep. shRNA expression was also observed in transgenic fibroblast cells TF-s2 and TF-s19 as positive controls.

To further confirm whether the shRNA inhibited MSTN expression in vivo, biceps brachii muscles were obtained by surgical biopsy from M17, M18, M23 and three control sheep, and MSTN precursor expression was detected by western blot analysis (Figure 2D). Compared with average expression levels of the controls, MSTN expression was reduced by about 61.12%, 31.32% and 10.11% in the transgenic M17, M18 and M23, respectively. This results suggested that MSTN expression was silenced by shRNA in transgenic sheep.

### Expression of MSTN-related Genes in Transgenic Sheep

**Figure 3:**
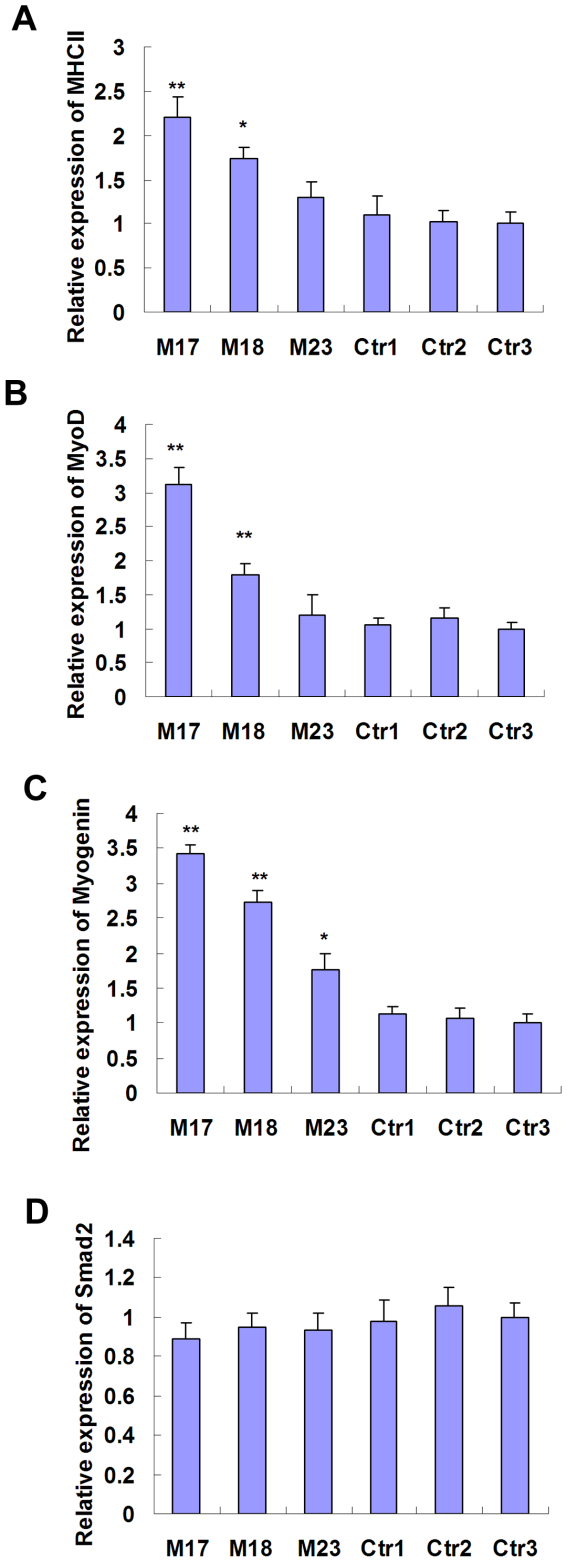
Expression levels of MHCII, MyoD, Myogenin and Smad2 in the muscle tissues of transgenic sheep. mRNA expression of MHCII (A), MyoD (B), Myogenin (C) and Smad2 (D) were determined using Real-time RT-PCR and normalized to GAPDH expression. * P<0.05, ** P<0.01.

We further determined whether shRNA-mediated MSTN knockdown affected expression of MSTN-related genes in transgenic sheep. Expression levels of MHCII, MyoD, Myogenin and Smad2 were analysed by Real-time RT-PCR. As indicated in Figure 3, expression levels of MHCII, MyoD and Myogenin in transgenic M17 and M18 were significantly higher than that of the controls. Expression levels of Smad2 were decreased in transgenic sheep, but without significant difference compared with the controls.

### Body Weight and Muscle Growth of Transgenic Sheep

**Figure 4:**
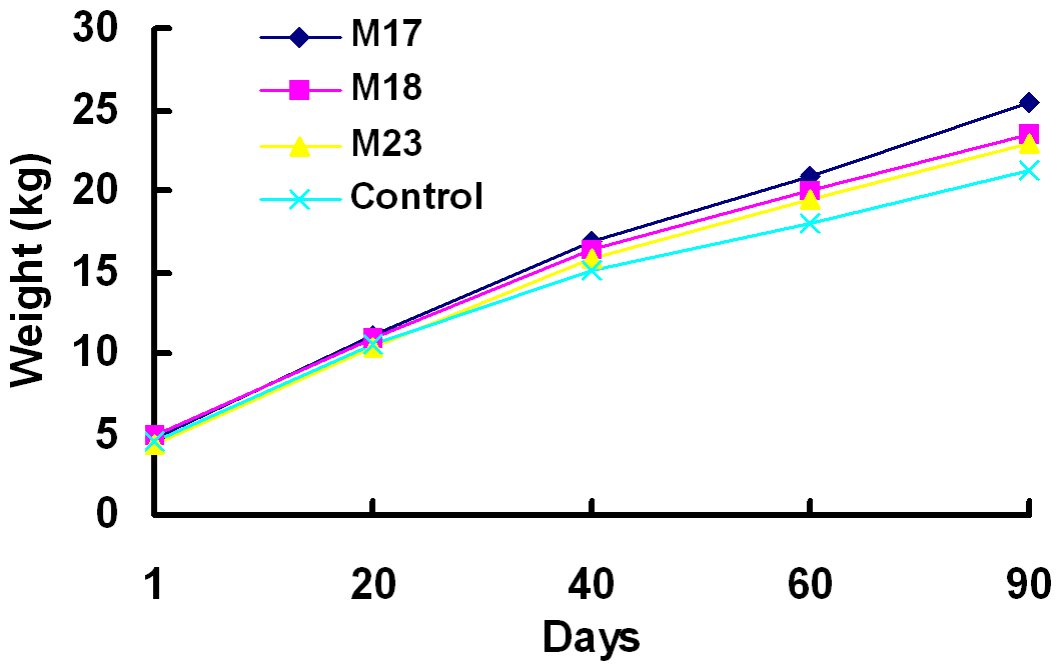
Body weight of transgenic sheep and controls. M17, M18, M23 and three control sheep were weighted at 1, 20, 40, 60 and 90 days after birth. Control values are average weights of three control sheep.

Based on body weight records, transgenic sheep showed a faster increase tendency in body weight than control sheep during the 90 days after birth (Figure 4). The 3-month-old M17 (25.42 kg), M18 (23.52 kg) and M23 (22.93 kg) were heavier than control sheep (21.22 kg, n = 3), respectively. Daily weight gain of M17 was 0.23 kg/d, when compared with 0.18 kg/d of control sheep.

**Figure 5:**
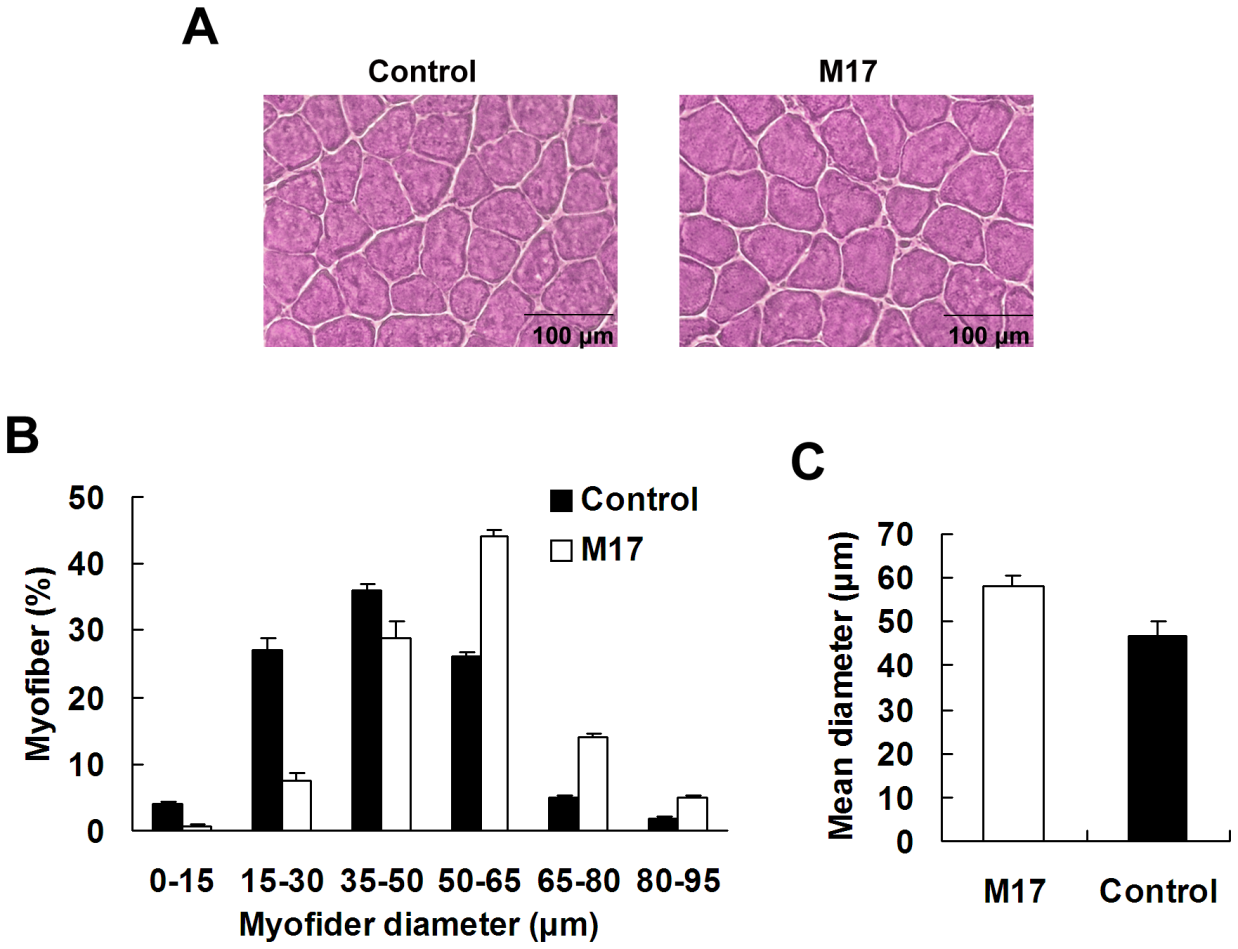
Morphometric analysis of muscles. (A) H&E staining displayed myofiber hypertrophy in muscles of M17 compared with control sheep. (B) Distribution of muscle fiber sizes in M17 and control sheep. A total of 500 fibers from each sheep were measured. (C) Mean myofiber diameter for M17 and controls.

Muscle tissues of M17 were isolated by surgical biopsy and sectioned for morphometric analysis. H&E staining showed larger myofiber sizes in the transgenic sheep (Figure 5A). Digital morphometric analysis of 500 myofibers from M17 muscle tissues revealed an increase in myofiber diameter compared with control sheep (Figure 5B). More myofibers were in the large myofiber diameter range (35–65 µm) compared with controls (Figure 5B). Mean diameter of M17 myofibers was 58 µm, whereas that of control myofibers was 47 µm (Figure 5C). No significant difference was found in the myofiber number per an unbiased 0.7 mm^2^ frame between M17 (912±13) and controls (869±16; n = 3). These results suggested that MSTN suppression may caused hypertrophy in transgenic sheep.

## Discussion

MSTN dysfunction resulted in dramatic increase of animal muscle mass due to hypertrophy and hyperplasia of muscle fibers [18]–[20]. Inhibition of MSTN expression by gene knockout or RNAi could promote the muscle growth and meat production of livestock animals. Due to the low efficiency of gene target in livestock animal somatic cells, RNAi is an ideal alternative for production of MSTN-knockdown transgenic livestock. In addition, there are some disadvantages to double-muscled cattle, including the reduction in female fertility, lower viability of offsprings, and delay in sexual maturation [21], [22]. The partial silencing of MSTN by RNAi in livestock may weaken some negative effect of null mutations, while at the same time increasing meat performance [23]. In the present study, we generated MSTN-knockdown transgenic sheep with increased muscle phenotype by RNAi and SCNT technology. To our knowledge, this is the first report in which MSTN-knockdown transgenic sheep were generated and showed increased muscle phenotype.

In our previous study, a shRNA expression cassette targeting MSTN had been constructed and induced significant decrease of MSTN expression by 90% in sheep fibroblasts [13]. This shRNA expression cassette included a sheep U6 and shRNA construct. Here this shRNA expression cassette was used to generate stable shRNA-expressing fibroblasts, and subsequently generate transgenic sheep by SCNT technology. shRNA expression was confirmed in muscles of transgenic sheep by RNase protection assay, indicating that the sheep U6 promoter could efficiently drive shRNA expression for gene silencing in vivo. However, in fact sheep M17 and M18 were derived from the same transgenic cell clones (TF-s19), but some variation in shRNA and MSTN expression levels were observed in muscles of M17 and M18 (Figure 2C, 2D). The possible reason may include: (1) Sheep U6 promoter may be influenced by epigenetic modifications as well as CMV and SV40 promoter hypermethylated in transgenic animal [24]–[26]. The hypermethylated CMV promoter probably resulted in silencing of transgene in animal cells [27], [28]; (2) Transgenic cell clones used for nuclear transfer may be mixed cell clones with different copies of shRNA gene integration. Different copies of shRNA resulted in variation of shRNA expression levels in transgenic sheep.

We transferred 429 embryos to the oviducts of 45 recipient sheep. Pregnancy rates were 24.4% at forty-five days after embryo transfer. Five lambs were born from five recipients. The clone efficiency was approximately 1.2%, consistent with other reports [29]. Transgenic sheep M21 and M24 died after birth, which may be as a result of developmental abnormalities of cloned fetus and placenta [30]. In addition, we did not see any abnormal development or behaviour in three live transgenic sheep up to at least six months of age. These results suggested that MSTN-knockdown may not affect development of cloned embryo and lamb.

Transgenic sheep M17, with largest reduction in MSTN expression levels (Figure 2D), showed a tendency to faster increase in body weight.than control sheep. Myofiber mean diameter of M17 was bigger than that of the non-transgenic controls, suggesting that MSTN-knockdown possibly caused hypertrophy of M17 myofiber. However, owing to our morphometric analysis of only one muscle biopsy from M17, further research is required to clarify whether MSTN-knockdown in sheep causes myofiber hyperplasia or hypertrophy and the effect on fiber types. The increased muscle mass in MSTN null mice and transgenic mice expressing high levels of the propeptide, follistatin, or a dominant negative form of activin receptor type IIB (ActRIIB) resulted from both hyperplasia and hypertrophy [17]–[19]. In contrast, missense mutant MSTN caused hyperplasia but not hypertrophy in mouse muscles, whereas dominant negative MSTN produced muscle hypertrophy without hyperplasia [31], [32]. These results indicated that this hypertrophic response and lack of hyperplasia may be due to the incomplete inhibition of MSTN gene expression.

We acknowledge that the present study only contained analysis of three transgenic sheep and growth performance of transgenic sheep need to be further observed. Work is currently underway to generate more MSTN-knockdown sheep by reclone transgenic sheep M17. In addition, application of transgenic animals for meat production is forbidden in some countries (e.g. European Union) and meat from cloned animals must be carefully assessed before entering the food chain. In summary, we successfully generated MSTN-knockdown transgenic sheep by RNAi and SCNT. Our findings demonstrate a promising approach to promoting muscle growth in livestock production.
